# Exploring representation of people without cohabiting carers in research on help-seeking for dementia diagnosis: a scoping review protocol

**DOI:** 10.3389/fpubh.2026.1927627

**Published:** 2026-09-22

**Authors:** Yixuan Zou, Sally Day, Lee-Fay Low

**Affiliations:** Sydney School of Health Sciences, Faculty of Medicine and Health, The University of Sydney, Sydney, NSW, Australia

**Keywords:** ageing and health, dementia, dementia diagnosis help-seeking, dementia help-seeking, geriatric medicine

## Abstract

**Background:**

People with possible dementia without cohabiting carers are a growing but under-researched group. Although help-seeking for dementia diagnosis often depends on the involvement of family members, carers, or other significant others, it remains unclear how living arrangement and supporter/carer-related variables are represented in this literature.

**Methods and analysis:**

This scoping review will be conducted according to Arksey and O’Malley’s methodological framework and reported in accordance with Preferred Reporting Items for Systematic Reviews and Meta-Analyses extension for Scoping Reviews (PRISMA-ScR). Searches will be conducted in Web of Science Core Collection, Medline, Embase, Scopus, APA PsycINFO, and CINAHL Ultimate. Eligible studies will be peer-reviewed primary research on real-life help-seeking for dementia diagnosis involving people experiencing cognitive concerns, memory problems, mild cognitive impairment, or dementia. Findings will be summarised and synthesised.

**Discussion:**

This review will map how living arrangement and supporter/carer-related variables are defined, reported, and analysed in research on help-seeking for dementia diagnosis. It is expected to identify important gaps in the current literature and inform recommendations for more consistent inclusion and reporting of these variables in future research.

**Ethics and dissemination:**

This scoping review protocol has been registered with the Open Science Framework (OSF) and is embargoed until 1 January 2027. The findings will be presented at relevant conferences, and the manuscript will be submitted to peer-reviewed journals.

**Clinical trial registration:**

https://osf.io/e32qu.

## Introduction

Globally, population ageing has brought renewed attention to how we support older people in our communities. The likelihood of a person living alone increases with age; data from Eurostat, the statistical office of the European Union, shows that nearly 20% of people aged 65 and over in the European Union live alone, a figure that continues to rise (1). In the United Kingdom, national data have also shown that around half of people aged 65 years and over live alone (2). According to the 2016 Australia Census, more than a quarter (25%) of all older people (aged 65 and over) living in private dwellings live alone (3). This trend is driven in high-income countries by sociodemographic factors including declining birth rates, increasing life expectancy, and evolving family structures (1). Cultural shifts towards individualism and the fragmentation of traditional community-based living arrangements as life expectancies increase have further increased the number of older people who live alone (4).

Dementia is also increasing globally; it is estimated that by 2050, more than 152 million people worldwide will be living with dementia (5). According to the Alzheimer’s Society, there are currently an estimated 982,000 people living with dementia in the UK, with projections indicating this will rise to 1.4 million by 2040 (2). The Australian Institute of Health and Welfare (AIHW) estimates that there were 425,000 Australians living with dementia in 2024 (6). This is equivalent to 16 people with dementia per 1,000 Australians. With an ageing and growing population, it is predicted that the number of Australians living with dementia will increase 2.5-fold, to over 1.1 million by 2065 (6). Dementia impairs cognition and also affects functional independence, mood, personality and behaviours (7). As the number of people living with dementia increases, the number of people with dementia who live alone or have limited supporter or carer availability is also projected to increase (8). People with dementia who live alone have compounded challenges related to cognitive impairment, ability to perform everyday tasks, and limited access to support (9). This group might be at higher risk due to missed medications, an inability to manage daily tasks, or difficulties seeking help during emergencies (10).

People with dementia who live alone constitute a growing but under-researched group. According to a statistical resource for U.S. data related to Alzheimer’s disease, approximately 65% of individuals with dementia live in the community, with 26% of them living at home alone (11). Despite growing global attention, existing dementia services may not always meet the needs of people with dementia who live alone, as they often presume the involvement of informal carers or family members (12). Studies have shown that people living alone are more likely to experience delayed diagnoses, poor care coordination, unmet health needs, and higher risks of hospitalisation (13, 14). This makes timely identification of dementia in this group particularly important, because earlier diagnosis allows crucial time for adjustment, decision making and future planning and access to guidance, financial support, and both pharmacological and nonpharmacological treatments (4). In contrast, delayed or missed diagnosis may contribute to unmet needs and poorer outcomes (15). Recognition of cognitive changes, validation of concerns, and progression towards diagnosis often depend on insight and actions of the person experiencing symptoms, as well as the observation and intervention of those around them (16). Some literature suggests that help-seeking for a dementia diagnosis is often initiated not by the person experiencing symptoms, but by family members, carers, or close friends (17–20). For people with dementia who live alone, this may reflect reduced awareness of cognitive decline, the normalisation of symptoms, or the fear and stigma associated with dementia, all of which may delay help-seeking (21).

Supporter or carer availability is not equivalent to living arrangement (22). Existing research describes informal caregiving as multidimensional, varying according to relationship, proximity, primacy, and the type of assistance provided. Contemporary definitions include both kin and non-kin supporters, and recognise that support may be provided by co-residing, nearby, or distance carers (23). Such support may also involve a broader caregiving network rather than a single individual (22). This suggests that living arrangement and supporter availability should not be treated as interchangeable - people who live alone may have substantial informal support, whereas those living with others may not necessarily have an identifiable carer or a person who is able to provide sufficient support. However, there is no clear consensus on how living arrangement and supporter availability should be defined, classified, or considered together in dementia research.

In this review, “people with possible dementia without cohabiting carers” refers to people seeking help for cognitive changes that may indicate dementia who do not have a co-residing informal carer able to support help-seeking. This includes people living alone, with varying support from non-cohabiting carers, and people sharing a home with someone unable to provide support.

Help-seeking for dementia diagnosis has been conceptualised in the Model of help-seeking for dementia diagnosis (Figure 1) as a non-linear pathway with four decision points, from recognising cognitive changes as a potential health problem, obtaining confirmation from family or friends, and deciding whether to seek medical help, through to GP facilitation of dementia assessment (24). This review therefore defines its target population by involvement in this help-seeking pathway rather than by a specific diagnostic status; relevant studies may include people with cognitive concerns, mild cognitive impairment, or suspected dementia. Given the limited research focused exclusively on help-seeking for dementia diagnosis among people with possible dementia without cohabiting carers, and the importance of significant others in recognising symptoms and initiating help-seeking, this review will examine how living arrangement and supporter/carer availability are described within the literature on help-seeking for dementia diagnosis. More specifically, it will explore how these variables are described and reported within the literature, and, if sufficient data are available, use the mapped evidence to inform a preliminary descriptive framework integrating living arrangement and supporter availability.

**Figure 1:**
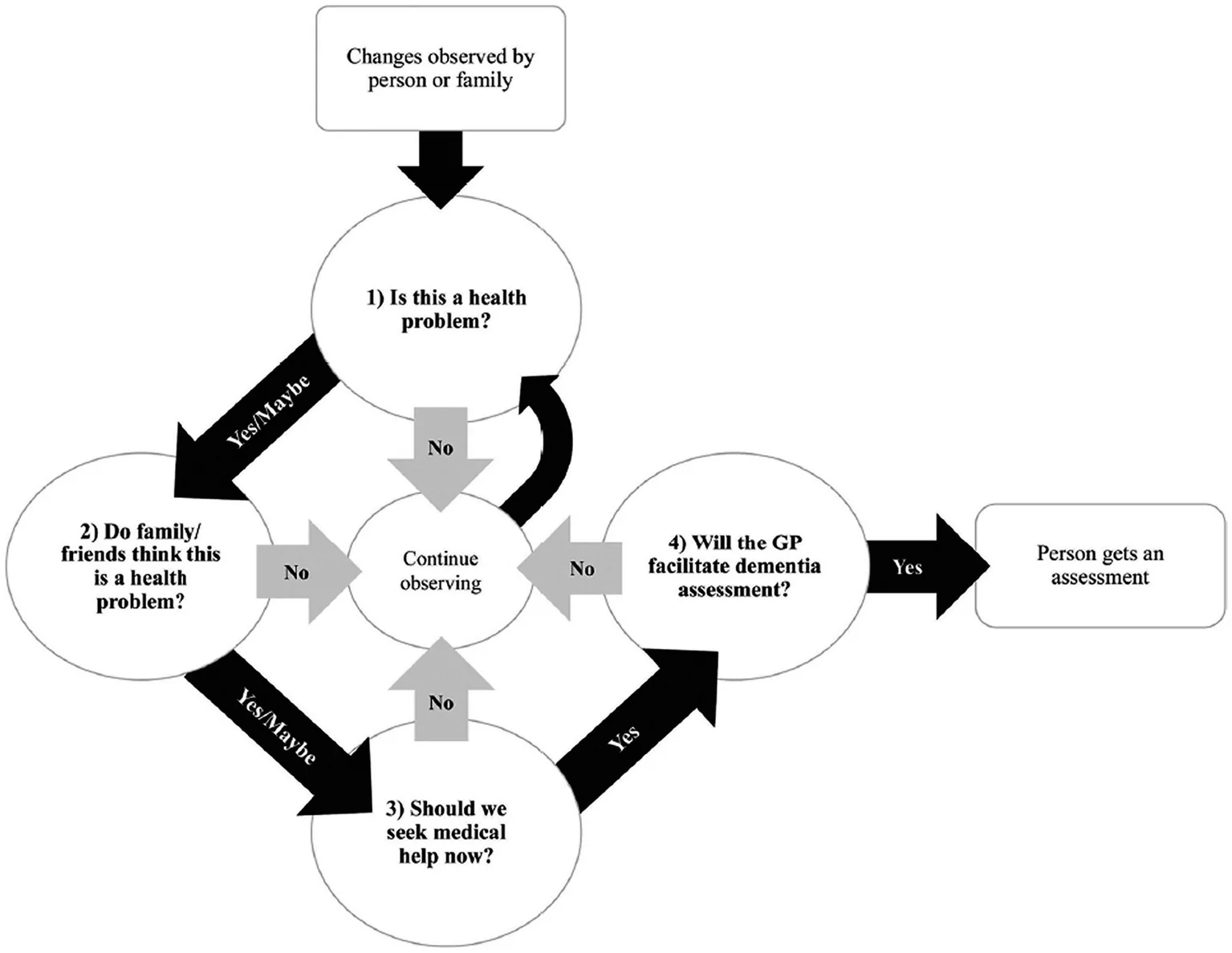
Model of help-seeking for dementia diagnosis (24).

## Aim and objectives

This scoping review aims to explore how people with possible dementia without cohabiting carers are represented within the literature on help-seeking for dementia diagnosis, with particular attention to how living arrangements and supporter/carer variables are defined, reported, and analysed. The specific objectives of this scoping review are to:

- Explore how living arrangement and carer/supporter related variables are defined and reported in studies on help-seeking for dementia diagnosis. Definitions might relate to kinship, geographical proximity, co-residence, and time spent with the person.
- Describe if and how living arrangement and supporter/carer availability are considered in analysis within research on help-seeking for dementia diagnosis.
- Synthesise any reported findings relating to living alone and supporter/carer availability in help-seeking for dementia diagnosis research.
- Explore whether the mapped evidence can inform a preliminary descriptive framework for characterising the relationship between living arrangement and supporter availability as related but distinct dimensions.

## Methods and analysis

### Trial registration

Trial registration: This scoping review protocol has been registered with the Open Science Framework (OSF) at https://osf.io/e32qu; the record is embargoed until 1 January 2027.

### Protocol design

This scoping review will be reported in accordance with the Preferred Reporting Items for Systematic Reviews and Meta-Analyses extension for Scoping Reviews (PRISMA-ScR) (25) (see Supplementary File A for PRISMA-ScR Checklist). The review will follow the five-stage framework outlined by Arksey and O’Malley (26).

The framework represents one of the most frequently utilised and published frameworks in the scoping review literature (27) and served as a guide for creating inclusion and exclusion criteria, the methodical search approach, the process of screening, the extraction of data, and the presentation of findings.

### Patient and public involvement

This scoping review protocol was shared with two dementia advocates and researchers, including one with experience of living alone with dementia and one with experience of supporting a family member living alone with dementia, for feedback prior to finalisation. Feedback was sought through a workshop-style meeting focused on the relevance, clarity, and design of the protocol, and informed subsequent revisions.

### Inclusion criteria

To be included, articles must satisfy the following four eligibility criteria:

- Collect data from people experiencing cognitive concerns, memory problems, mild cognitive impairment, or dementia, or their informal supporters.
- Focus on investigating real-life experiences of help-seeking for dementia diagnosis.
- Be original empirical studies based on primary data, published in peer-reviewed journals.
- English-language articles published from 2006 onwards.

### Exclusion criteria

- Participants with cognitive problems attributable to specific conditions or causes other than dementia, or informal carers of such participants
- Hypothetical help-seeking intentions
- Studies focused solely on post-diagnostic support, service use, or communication of a diagnosis.
- Editorials, opinion pieces, letters, conference abstracts, theses, government reports, industry or organisation reports, and other grey literature or non-empirical publications.

Eligible experiences will correspond to at least one decision point in the Model of help-seeking for dementia diagnosis (24), irrespective of participants’ diagnostic status at data collection. Retrospective accounts, including those from people subsequently diagnosed with dementia, and studies of service access or assessment are eligible when they describe this help-seeking pathway. Where actual experiences and hypothetical intentions are combined, the actual experiences must be separately extractable.

Although diagnostic criteria and technologies continue to evolve, guidelines for comprehensive dementia evaluation have existed for more than 20 years (11). Studies published from 2006 onwards were therefore included to capture help-seeking research conducted within a broadly comparable diagnostic context while maintaining a feasible scope.

### Information sources

A comprehensive literature search will be undertaken to identify potentially relevant studies. The search is expected to commence in July 2026. Planned bibliographic databases include: Web of Science Core Collection, Medline (Ovid), Embase (Ovid), Scopus, APA PsycINFO (Ovid), and CINAHL Ultimate. These databases were selected to capture literature across biomedical, psychological, and multidisciplinary health research relevant to help-seeking for dementia diagnosis. Grey literature was excluded because this review focuses on how living arrangement and supporter/carer availability are represented in peer-reviewed empirical research. Limiting eligibility to peer-reviewed primary studies was intended to support more consistent extraction and comparison of study variables, methods, and analyses (28, 29).

### Search strategy

The search strategy has been developed collaboratively by the research team, including an experienced psychologist, epidemiologist, and dementia researcher (LFL) with extensive experience in conducting systematic reviews and expertise in dementia, aged care, and intervention research involving older people; an occupational therapist and dementia researcher (SD) with extensive clinical experience in health and aged care, as well as expertise in dementia and implementation research; and a PhD researcher in medicine and health at the University of Sydney (YZ). The search strategy has also been reviewed and refined in consultation with an academic engagement librarian at the University of Sydney.

The search has been structured around two main concepts: dementia and help-seeking. Living arrangement and supporter-related terms were not included because these variables may be reported only in participant descriptions rather than being the focus of a study. Search terms have been developed iteratively and refined to balance sensitivity and specificity in relation to the review objectives. During piloting, the team checked that studies already known to meet the help-seeking criteria were retrieved by the search. Please see Supplementary File B for the complete search terms for each database.

### Selection of sources

Records from all results will be imported into the EndNote 21 reference management tool (30). Duplicates that are identified by matching the DOI or title, author, year, and journal will be excluded. The citations will subsequently be collated and reviewed using Covidence systematic review software (31). Two reviewers (YZ, and either LFL or SD) will independently screen records based on title and abstract using the eligibility criteria. Conflicts will be resolved through team discussion. Full-text screening will be conducted independently by the two groups of reviewers, and any conflicts will be resolved in the same way as in the abstract screening stage.

### Charting the data

A data-charting form will be developed in Excel to extract relevant information from the included studies. Supplementary File C for Data Charting Table presents the data extraction items. Living arrangement and supporter availability will be charted as related but distinct dimensions, with categories refined iteratively as additional patterns emerge from the included studies. The dimensions charted will include co-habitation, kinship, geographic proximity, frequency of contact, availability during health-related decision-making, caregiving role, and the type and intensity of support. Roles may include observing changes, raising concerns, practical help, legal responsibility, and capacity to increase support as needs change. Ambiguous terminology will be retained as reported and discussed within the team. Insufficiently reported information will be coded as “not reported” rather than taken to indicate absence of support.

The data-charting form will be refined during the review process if additional relevant categories emerge. The charting form will first be piloted on a small number of included studies to ensure consistency in interpretation and extraction.

Data extraction will then be completed by one reviewer (YZ), with all extracted fields checked by a second reviewer (LFL or SD) for an initial sample of at least 10% of studies. Any uncertainties or disagreements will be resolved through discussion within the research team to ensure consistency in interpretation of the items. Additional studies will be checked until consistency data extraction ensured. For mixed samples, living arrangement and supporter-related information will be extracted at the subgroup level where separately reported. Multiple publications from the same dataset will be linked as related reports and counted as one study in study-level summaries. Variables reported only in demographic tables will be recorded as reported, but not as analysed unless they are explicitly included in the study analysis.

Consistent with scoping review methodology all articles that satisfy our inclusion criteria will be included, irrespective of their methodological quality (32).

### Collating, summarising and reporting the results

The results of the review will be collated and summarised using descriptive summary and narrative synthesis. A PRISMA flow diagram will be used to report the study selection process.

The descriptive summary will be used to present the characteristics of the included studies and to summarise the extent to which living arrangement and supporter/carer variables were reported and considered across the literature. We will report the number and percentage of unique included studies that report living arrangement, report supporter availability, use each variable in analysis, or present subgroup findings. Percentages will use all unique included studies. Narrative synthesis will be used to summarise how these variables were defined and described, then to synthesise any reported findings relevant to living alone or supporter/carer availability. Reported terminology will be retained and grouped descriptively where useful.

## Discussion

This scoping review will provide insight into how living arrangement and supporter/carer availability are represented in research on help-seeking for dementia diagnosis. By mapping how these variables are defined, reported, and incorporated into analysis, the review will identify important gaps in the current literature, both in the inclusion of people with possible dementia without cohabiting carers in dementia research and in the reporting of these variables. Furthermore, if sufficient data are available, it may inform a preliminary descriptive framework that treats living arrangement and supporter availability as related but distinct dimensions. The findings will guide recommendations for defining and reporting of these variables in future research, and may also lead to the development of more inclusive approaches to dementia diagnosis research.

Potential challenges include a large volume of retrieved studies and inconsistent or incomplete reporting of living arrangement and supporter availability, which may complicate data charting and limit subgroup comparisons or the development of a preliminary descriptive framework. The date, English-language, and peer-reviewed publication restrictions may also exclude relevant evidence. These limitations will be considered when interpreting the review findings.

## References

[1] Puyané M Chabrera C Camón E Cabrera E. Uncovering the impact of loneliness in ageing populations: a comprehensive scoping review. BMC Geriatr. (2025) 25:244. doi: 10.1186/s12877-025-05846-4, 40211165 PMC11984289

[2] Gamble LD Clare L Martyr A Caulfield M Charlwood C Hulme C . Prevalence of living alone with dementia and other progressive neurological conditions: findings from primary care data in England. BMC Med. (2025) 23:607. doi: 10.1186/s12916-025-04443-x, 41188892 PMC12584314

[3] Australian Institute of Health and Welfare (2024) Older australians. Australian Institute of Health and Welfare Available online at: https://www.aihw.gov.au/reports/older-people/older-australians/contents/housing-and-living-arrangements (Accessed June 06, 2026).

[4] Rasmussen J Langerman H. Alzheimer's disease—why we need early diagnosis. Degener Neurol Neuromuscul Dis. (2019) 9:123–30. doi: 10.2147/dnnd.S228939, 31920420 PMC6935598

[5] GBD 2019 Dementia Forecasting Collaborators. Estimation of the global prevalence of dementia in 2019 and forecasted prevalence in 2050: an analysis for the global burden of disease study 2019. Lancet Public Health. (2022) 7:e105–25. doi: 10.1016/s2468-2667(21)00249-8, 34998485 PMC8810394

[6] Australian Institute of Health and Welfare (2025) Dementia in Australia Available online at: https://www.aihw.gov.au/reports/dementia/dementia-in-aus/contents/summary (Accessed June 06, 2026).

[7] Dubbelman MA Jutten RJ Tomaszewski Farias SE Amariglio RE Buckley RF Visser PJ . Decline in cognitively complex everyday activities accelerates along the Alzheimer’s disease continuum. Alzheimer's Res Ther. (2020) 12:138. doi: 10.1186/s13195-020-00706-2 PMC7597034 33121534

[8] Clare L Martyr A Henderson C Gamble L Matthews FE Quinn C . Living alone with mild-to-moderate dementia: findings from the IDEAL cohort. J Alzheimer's Dis. (2020) 78:1207–16. doi: 10.3233/jad-200638, 33104029

[9] Crance SJ Yu F. Characteristics, needs, and perspectives of individuals living alone with dementia: an integrative review. Health Sci Rep. (2025) 8:e70348. doi: 10.1002/hsr2.70348, 39867711 PMC11757279

[10] McKhann GM Knopman DS Chertkow H Hyman BT Jack CR Jr Kawas CH . The diagnosis of dementia due to Alzheimer's disease: recommendations from the National Institute on Aging-Alzheimer's Association workgroups on diagnostic guidelines for Alzheimer's disease. Alzheimers Dement. (2011) 7:263–9. doi: 10.1016/j.jalz.2011.03.005, 21514250 PMC3312024

[11] Alzheimer's Association. 2024 Alzheimer's disease facts and figures. Alzheimers Dement. (2024) 20:3708–821. doi: 10.1002/alz.13809, 38689398 PMC11095490

[12] Clare L Martyr A Gamble L Caulfield M Charlwood C Hulme C . Living alone with dementia is a neglected source of inequality: findings from a scoping review. Innov Aging. (2025) 9:igaf122.884. doi: 10.1093/geroni/igaf122.884, 41372938 PMC12817627

[13] Clare L Martyr A Caulfield M Gamble LD Charlwood C Ward J . Living alone with dementia is a neglected source of inequality: findings from a scoping review of research evidence. Syst Rev. (2025) 15:16. doi: 10.1186/s13643-025-03002-y, 41372938 PMC12817627

[14] Polack S Bell G Silarova B Hebditch M Tingle A Sommerlad A . Non-pharmacological interventions for people with dementia who live alone: a systematic review. Int J Geriatr Psychiatry. (2025) 40:e70059. doi: 10.1002/gps.70059, 40011198 PMC11864918

[15] Molvik I Kjelvik G Selbæk G Rokstad AMM. The significance of a dementia diagnosis from the perspective of the family caregivers: a qualitative study. BMC Health Serv Res. (2025) 25:487. doi: 10.1186/s12913-025-12657-1, 40176028 PMC11963315

[16] Shaw BA Yang T-C Kim S. Living alone during old age and the risk of dementia: assessing the cumulative risk of living alone. J Gerontol B Physcol Sci Soc Sci. (2023) 78:293–301. doi: 10.1093/geronb/gbac156, 36179214 PMC9938918

[17] Parker M Barlow S Hoe J Aitken LM. The bubble of normalisation: a qualitative study of Carers of people with dementia who do not seek help for a diagnosis. J Geriatr Psychiatry Neurol. (2022) 35:717–32. doi: 10.1177/08919887211060018, 34951319 PMC9386763

[18] Perry-Young L Owen G Kelly S Owens C. How people come to recognise a problem and seek medical help for a person showing early signs of dementia: a systematic review and meta-ethnography. Dementia. (2018) 17:34–60. doi: 10.1177/1471301215626889, 26764265 PMC5758935

[19] Phillipson L Magee C Jones S Reis S Skaldzien E. Dementia attitudes and help-seeking intentions: an investigation of responses to two scenarios of an experience of the early signs of dementia. Aging Ment Health. (2015) 19:968–77. doi: 10.1080/13607863.2014.995588, 25554920

[20] Rogers K Coleman H Brodtmann A Darby D Anderson V. Family members’ experience of the pre-diagnostic phase of dementia: a synthesis of qualitative evidence. Int Psychogeriatr. (2017) 29:1425–37. doi: 10.1017/S1041610217000862 28587690

[21] Parker M Barlow S Hoe J Aitken L. Persistent barriers and facilitators to seeking help for a dementia diagnosis: a systematic review of 30 years of the perspectives of carers and people with dementia. Int Psychogeriatr. (2020) 32:611–34. doi: 10.1017/S1041610219002229, 32024558

[22] Happ MB Moss KO Dabbs AD Narby E Song MK. Defining informal caregiving and caregivers for persons living with dementia. J Gerontol Nurs. (2024) 50:41–5. doi: 10.3928/00989134-20241118-05, 39621519

[23] Wosko P Pfabigan J Trukeschitz B Reitinger E Pichler B Pleschberger S. Nonkin Carers' roles and contributions to the support of older people living alone: an analysis of qualitative data. J Gerontol B Psychol Sci Soc Sci. (2024) 79. doi: 10.1093/geronb/gbae008, 38285494 PMC10998341

[24] Low LF Barcenilla-Wong A Laver K Yates M Gibson C Shen S . Development of a model of help-seeking for dementia diagnosis by the person experiencing changes and family supporters. Aging Ment Health. (2024) 29:814–23. doi: 10.1080/13607863.2024.2430537, 39578958

[25] Tricco AC Lillie E Zarin W O'Brien KK Colquhoun H Levac D . PRISMA extension for scoping reviews (PRISMA-ScR): checklist and explanation. Ann Intern Med. (2018) 169:467–73. doi: 10.7326/m18-0850 30178033

[26] Arksey H O'Malley L. Scoping studies: towards a methodological framework. Int J Soc Res Methodol. (2005) 8:19–32. doi: 10.1080/1364557032000119616

[27] Peters MDJ Marnie C Colquhoun H Garritty CM Hempel S Horsley T . Scoping reviews: reinforcing and advancing the methodology and application. Syst Rev. (2021) 10:263. doi: 10.1186/s13643-021-01821-3, 34625095 PMC8499488

[28] Ford TN Hanneke R Ezeani T Mitchell UA. Measuring subjective well-being for people racialised as black in the USA: a scoping review protocol. BMJ Open. (2026) 16:e110875. doi: 10.1136/bmjopen-2025-110875, 41927294 PMC13052548

[29] Zou Y Sharpe A Demant D. The effectiveness of podcasts in promoting health among young people: a scoping review. Health Promot Int. (2025) 40:daaf102. doi: 10.1093/heapro/daaf102, 40607512 PMC12223501

[30] Clarivate (2023) EndNote - The best citation & reference management tool. Clarivate EndNote. Available online at: https://endnote.com (Accessed June 10, 2026).

[31] Covidence. (2020) Covidence - Better systematic review management. Covidence. Available online at: https://www.covidence.org (Accessed June 10, 2026).

[32] Tricco AC Lillie E Zarin W O'Brien K Colquhoun H Kastner M . A scoping review on the conduct and reporting of scoping reviews. BMC Med Res Methodol. (2016) 16:15. doi: 10.1186/s12874-016-0116-4 PMC4746911 26857112

