# Supplementary material for: Exploring representation of people without cohabiting carers in research on help-seeking for dementia diagnosis: a scoping review protocol
Source: Front Public Health. 2026 Sep 22;14:1927627. doi: 10.3389/fpubh.2026.1927627 (PMC13638369; doi:10.3389/fpubh.2026.1927627)
Supplement: Supplementary file 2 [file Table_2.DOCX]

| **Database** | **Search Strategy (English language and date limits applied)** |
| --- | --- |
| Web of Science Core Collection | (dement* OR Alzheimer* OR "MCI") (Topic) or cognitiv* NEAR/2 (declin* or impair* or deteriorat* or dysfunction*) (Topic) or memory NEAR/2 (declin* or impair* or deteriorat* or dysfunction* or concern*) (Topic)  AND help* NEAR/2 seek* (Topic) or seek* NEAR/1 medical (Topic) or pathway NEAR/3 diagnos* (Topic) or time NEAR/3 diagnos* (Topic) |
| CINAHL Ultimate | MH "Dementia+" OR MH "Dementia Patients" OR (MH "Mild Cognitive Impairment" OR MH "Cognition Disorders") OR MH "Memory Disorders" OR XB (dement* OR Alzheimer* OR "MCI") OR XB (cognitiv* N2 (declin* OR impair* OR deteriorat* OR dysfunction*)) OR XB (memory N2 (declin* OR impair* OR deteriorat* OR dysfunction* OR concern*))) AND (MH "Help Seeking Behavior" OR XB (help* N2 seek*) OR XB (seek* N1 medical) OR XB (pathway N3 diagnos*) OR XB (time N3 diagnos*)) |
| Scopus | ( ( TITLE-ABS-KEY ( dement* OR alzheimer* OR "mci" ) OR TITLE-ABS-KEY ( cognitiv* W/2 ( declin* or impair* or deteriorat* or dysfunction* ) ) OR TITLE-ABS- KEY ( memory W/2 ( declin* or impair* or deteriorat* or dysfunction* or concern* ) ) ) ) AND (( TITLE-ABS-KEY ( help* W/2 seek* ) OR TITLE-ABS-KEY ( seek* W/1 medical ) OR TITLE-ABS-KEY ( pathway W/3 diagnos* ) OR TITLE-ABS-KEY ( time W/3 diagnos* ) )) |
| Medline | (exp Dementia/ OR Cognitive Dysfunction/ OR Memory Disorders/ OR (dement* OR Alzheimer* OR "MCI").ti,ab,kf. OR (cognitiv* adj2 (declin* OR impair* OR deteriorat* OR dysfunction*)).ti,ab,kf. OR (memory adj2 (declin* OR impair* OR deteriorat* OR dysfunction* OR concern*)).ti,ab,kf.) AND (help-seeking behavior/ OR (help* adj2 seek*).ti,ab,kf. OR (seek* adj1 medical).ti,ab,kf. OR (pathway adj3 diagnos*).ti,ab,kf. OR (time adj3 diagnos*).ti,ab,kf.) |
| Embase | (dementia/ OR "behavioral and psychological symptoms of dementia"/ OR corticobasal degeneration/ OR diffuse neurofibrillary tangles with calcification/ OR early onset dementia/ OR exp frontotemporal dementia/ OR mental deterioration/ OR mixed dementia/ OR "mixed depression and dementia"/ OR multiinfarct dementia/ OR presenile dementia/ OR primary familial brain calcification/ OR pseudodementia/ OR exp senile dementia/ OR cognitive defect/ OR mild cognitive impairment/ OR memory disorder/ OR (dement* OR Alzheimer* OR "MCI").ti,ab,kf. OR (cognitiv* adj2 (declin* OR impair* OR deteriorat* OR dysfunction*)).ti,ab,kf. OR (memory adj2 (declin* OR impair* OR deteriorat* OR dysfunction* OR concern*)).ti,ab,kf.) AND (help seeking behavior/ OR (help* adj2 seek*).ti,ab,kf. OR (seek* adj1 medical).ti,ab,kf. OR (pathway adj3 diagnos*).ti,ab,kf. OR (time adj3 diagnos*).ti,ab,kf.) |
| APA PsycINFO | (exp Dementia/ OR Dementia Patients/ OR Mild Cognitive Impairment/ OR Cognition Disorders/ OR Memory Disorders/ OR (dement* OR Alzheimer* OR "MCI").ti,ab. OR (cognitiv* adj2 (declin* OR impair* OR deteriorat* OR dysfunction*)).ti,ab. OR (memory adj2 (declin* OR impair* OR deteriorat* OR dysfunction* OR concern*)).ti,ab.) AND (Help Seeking Behavior/ OR (help* adj2 seek*).ti,ab. OR (seek* adj1 medical).ti,ab. or (pathway adj3 diagnos*).ti,ab,id. or (time adj3 diagnos*).ti,ab,id.) |
|  |  |
